# Integrated transcriptome and metabolome analysis of photoperiod effects on testosterone secretion in ChaHua chicken No.2 Roosters

**DOI:** 10.1016/j.psj.2025.104916

**Published:** 2025-02-17

**Authors:** Mengqian Liu, Lei Tan, Xinlu Li, Haojie Li, Yong Zhang, Xiannian Zi, Changrong Ge, Kun Wang

**Affiliations:** a College of Animal Science and Technology, Yunnan Agricultural University, Yunnan 650201, China; b Key Laboratory of Feed Biotechnology, Institute of Feed Research, Chinese Academy of Agricultural Sciences, Beijing 100081, China; c College of Animal Science and Technology, Gansu Agricultural University, Gansu 730070, China

**Keywords:** Transcriptome, Metabolome, light intensity, Chahua No.2 rooster, Testosterone

## Abstract

This study investigated the impact of light duration on Testosterone secretion in Chahua No.2 roosters, utilizing combined Transcriptome and Metabolome analyses to uncover critical genes, Metabolites, and signaling pathways. We randomly selected 240 Chahua No.2 roosters at 42 days old and divided them into four groups: simulated natural light (Ⅰ), 12L:12D (Ⅱ), 16L:8D (Ⅲ), and 20L:4D (Ⅳ), each group has 6 replicates, with 10 chickens per replicate. Blood samples were collected at 91 and 140 days post-hatch to measure Testosterone levels. Results showed that at 91 days, group Ⅳ had significantly higher Testosterone levels than groups Ⅰ, Ⅱ, and Ⅲ (*P* < 0.01), with group Ⅲ also higher than groups Ⅰ (*P* < 0.01) and Ⅱ (*P* < 0.05). By 140 days, group Ⅳ maintained significantly higher Testosterone than groups Ⅰ and Ⅱ (*P* < 0.01) and higher than group Ⅲ (*P* < 0.05), while group Ⅲ was also elevated compared to groups Ⅰ and Ⅱ (*P* < 0.05). Testicles Transcriptomics analysis revealed 891 differentially expressed genes, including 479 down-regulated and 412 up-regulated genes. Key signaling pathways identified included Steroid Hormone Biosynthesis, Cytochrome P450, and retinol metabolism. Testicles Metabolomics analysis identified 174 differential Metabolites, with 91 up-regulated and 83 down-regulated, focusing on pathways like Amino Sugar Metabolism and Tryptophan Metabolism. Integrated analysis pinpointed 19 common signaling pathways, with the top ten including Cytochrome P450, tyrosine metabolism, and amino acid biosynthesis. Our findings indicate that extending light duration enhances Testosterone secretion in roosters. Through comprehensive transcriptomic and metabolomic analyses, we established that pathways associated with steroid hormone synthesis and Cytochrome P450 play a crucial role in light duration-regulated Testosterone secretion, highlighting key genes such as C*YP11A1, CYP17A1*, and *HSD3B1*, alongside Metabolites like ergosterol-5,7,22,24(28)-tetraene-3beta-alcohol.

## Introduction

Light is an important environmental factor influencing Testosterone secretion in Roosters, and its effects are primarily mediated through light receptors in the Retina and Hypothalamus (Bédécarrats, 2015). Photoreceptors, when stimulated by light, generate nerve impulses that are transmitted to the Hypothalamus, thereby promoting the release of Gonadotropin-Releasing Hormone (GnRH). GnRH acts on the Pituitary Gland, stimulating the secretion of Follicle-Stimulating Hormone (FSH) and Luteinizing Hormone (LH). These hormones work synergistically on the testes, promoting Testosterone secretion by the interstitial cells. Testosterone, a steroid hormone, is synthesized through a series of enzymatic reactions involving Cytochrome P450 mixed-function oxidases or members of the Hydroxysteroid Dehydrogenase family. The synthesis of steroids is mainly regulated by the LH/cAMP-PKA pathway (Xu et al., 2015), where LH stimulates the conversion of cholesterol to Testosterone in the interstitial cells of the testes (Xu et al., 2014). Testosterone secretion serves as a key indicator for evaluating the development of Roosters' gonads and is primarily secreted by the testes (Smith et al., 2015). Previous studies have shown that prolonged light exposure can increase Testosterone levels in Roosters, with a positive linear correlation between light duration and Testosterone secretion (Renden et al., 1991). Moreover, extended light exposure promotes the development of the gonads in Roosters (Li et al., 2015).

In recent years, there has been increasing research on the impact of light duration on the reproductive performance of Chickens, particularly regarding how different light cycles affect testicular development, sexual maturity, and hormone secretion. Numerous studies have indicated that light duration significantly influences poultry reproductive performance. For example, a study by Tang (2013) on the development of 90-day-old Yellow-Feather Broilers found that Roosters exposed to 16 h of light had heavier testes than those exposed to 23 h of light. Similarly, Shi et al. (2021) reported that prolonged light exposure significantly increased testicular weight in White Leghorn and Beijing You Chickens. However, Floyd and Tyler (2011) observed that in Cobb Broilers, testicular weight gradually decreased after 20 weeks of age as light duration increased. Additionally, Yang et al. (1998) found that male Turkeys exposed to long-day light cycles matured earlier and exhibited increased concentrations of LH and Testosterone from 10 or 12 weeks of age. Research on monochromatic light has also explored how various light cycles affect testicular development, sexual maturity, and hormone secretion in Roosters. For example, Shen et al. (2016) found that White Artificial Light was more effective than Red and Blue Artificial Light in maintaining semen quality in male Geese. Another study demonstrated that Red and Yellow Light positively influenced the reproductive performance and sperm quality of Dandarawi Roosters, while Green Light had adverse effects (Sayed and Galal, 2018). Moreover, studies comparing constant light cycles with extended light or monochromatic light exposure have also gained attention. Charles et al. (1992) showed that extended light exposure could stimulate sexual development in Meat Chicks as early as 7 weeks of age. Bartman et al. (2021) found that Targeted Differential Photostimulation (TDP) using long-day Red Light and short-day Green Light significantly improved reproductive performance in Roosters, reflected in increased semen volume, sperm vitality, sperm concentration, and testicular weight.

In conclusion, while numerous studies have addressed the effects of different light cycles and light types on Rooster reproductive development and hormone secretion, research on the molecular mechanisms by which light duration affects Testosterone secretion in the testes remains limited. This study focuses on the Chahua No.2 Rooster breed, aiming to investigate the impact of light duration on Testosterone secretion. By integrating testicular transcriptomic and metabolomic analyses, this study will identify the key genes, Metabolites, and signaling pathways influenced by light duration in the Testosterone secretion of Chahua No.2 Roosters. This research will provide new scientific insights into the molecular regulatory mechanisms underlying the effects of light duration on Testosterone secretion in Chahua No.2 Roosters, advancing Poultry Genetic Breeding Technology and laying the foundation for breeding high-quality, high-yield poultry breeds.

## Materials and methods

### Experimental animals and feeding management

The Chahua No.2 roosters used in this study were sourced from the Houshan Practice Chicken Farm at Yunnan Agricultural University. Healthy breeding hens were artificially inseminated, and Eggs were collected and incubated. After hatching, the chicks were raised until the 42nd day, at which point the brooding temperature withdrawal process was completed. A total of 240 healthy Chahua No.2 roosters, all aged 42 days, were randomly selected and raised until they reached 140 days of age. Except for variations in light exposure, the feeding environment, management practices, and nutritional feed remained consistent throughout the study. Vaccinations were administered on time, following the established immunization schedule.

### Dietary composition and nutrient level

The experimental feed consisted of a specially formulated diet tailored in stages to match the specific traits of high-quality Yunnan local chickens. The dietary composition and nutrient levels for the brooding and growing phases are detailed in Table 1.

**Table 1:** Dietary composition and nutrient level

Formulation/%	Brood time	Breed time	Nutrition level/%	Breed time	Brood time
Maize	61.20	60.90	Metabolic energy/(MJ/kg)	11.63	12.13
Soybean meal	30.16	25.22	Crude protein	15.50	19.30
Fish meal	3.60	0.00	Calcium	0.80	0.85
Wheat bran	0.00	10.00	Availed phosphorus	0.37	0.37
Soybean oil	1.10	0.00	Lysine	0.79	0.98
Cahpo_4	1.50	1.50	Methionine	0.36	0.43
Fine stone powder	0.70	0.60			
Middling flour	0.41	0.46			
Methionine	0.08	0.07			
Salt	0.25	0.25			
Premix	1.00	1.00			
Total	100.00	100.00			

Note: The premix supplies the following per kilogram of the diet: V_A_ 15,000 U, V_D3_ 3,300 U, V_E_ 62.5 mg, V_K_ 3.6 mg, V_B_13 .0 mg, V_B2_ 9 .0 mg, V_B6_ 6.0 mg, V_B12_0.03 mg, folic acid 60 mg, nicotinic acid 60 mg, pantothenic acid 18 mg, biotin 0.36 mg,choline chloride 600 mg, Se 0.15 mg, I 0.35 mg, Cu 12 mg, Mn 60 mg, Fe 80 mg, Zn 75 mg, along with antibacterial growth promoters, antioxidants, etc. The metabolic energy is a calculated value, while the remaining values are determined.

### Experiment design

A total of 240 Chahua No.2 roosters were randomly assigned to four groups, each with six replicates and ten roosters per replicate. They were housed in cages with a controlled feeding environment and consistent feed standards. The study established four groups, each exposed to different light patterns. Starting from day 43, the light intensity remained constant at 10 lx. The light cycles of each group were set as simulated natural light, 12L:12D, 16L:8D, and 20L:4D, respectively. The simulated natural light cycle correlated with the daily sunrise and sunset times in Kunming, while the other three cycles commenced at 6:00 AM. The groups were designated as I, II, III, and IV, respectively.

### Measurement indexes and methods

Blood samples were collected from the subwing vein of 10 chickens from each group at 91 and 140 days of age, respectively. Serum was separated (3000 rpm, 15 min, 4 C) and stored at −20 °C for Testosterone (T) determination analysis. A double antibody one-step sandwich enzyme-linked immunosorbent assay (ELISA) kit (Baolai Bio, Jiangsu, China)facilitated the T content determination.

### Sample collection

Testicular tissue was collected at slaughter at 140 days of age, frozen, and stored at −80 C. The two most significantly different groups of samples (i.e. groups I and IV) were sent to the company for a combined Transcriptome and Metabolome analysis based on the Testosterone content. Four tissue samples were sent to each group for Transcriptome sequencing. Five tissue samples were sent to each group for Metabolome sequencing.

### Total RNA extraction and transcriptome sequencing of testicular tissue

Total RNA was extracted from testicular tissue using TRIzol Reagent (Life Technologies, California, USA) following the manufacturer's protocol. RNA concentration and purity were assessed using the NanoDrop 2000 (Thermo Fisher Scientific, Wilmington, DE). The integrity of the RNA was evaluated using the RNA Nano 6000 assay kit on the Agilent Bioanalyzer 2100 system (Agilent Technologies, CA, USA). After confirming sample quality, cDNA libraries were constructed and sequenced in PE150 mode on the Illumina NovaSeq 6000 sequencing platform. Sequencing was performed by Baimaike Biotechnology Co., Ltd. Raw data refinement involved the elimination of sequences containing adapters, poly-N, and low-quality reads (those with an N removal ratio exceeding 10 %). Reads were discarded if more than 50 % had quality values of *Q* ≤ 10, resulting in clean data. The cleaned reads were aligned to the reference genome (GRCg6a) using HISAT2 (Kim et al., 2015). StringTie (Pertea et al., 2015) was then used for read comparison, assembly, and reconstruction, while gene expression levels were quantified using the FPKM (Trapnell et al., 2010) method.

### Metabolomics assays of testicular tissue

A total of 50 mg testicular tissue samples were precisely weighed. Subsequently, 1000 μL of an extract containing the internal standard composed of a methanol-acetonitrile-water mixture in a 2:2:1 ratio, with an internal standard concentration of 20 mg/L-was added. The mixture was vortexed and agitated for 30 s. Steel balls were incorporated, followed by treatment in a 45 Hz grinder for 10 min, with an additional 10 min sonication in an ice water bath. The liquid chromatography-mass spectrometry system used for metabolomics analysis consisted of a Waters UPLC Acquity I-Class PLUS (Waters, USA), coupled with a Waters Xevo UPLC G2-XS QTOF (Waters, USA), using a 1 μL injection volume. The chromatographic column employed was the Acquity UPLC HSS T3 column (1.8 μm, 2.1 × 100 mm, Waters, USA). The mobile phase for positive and negative ionization modes included mobile phase A: a 0.1 % formic acid aqueous solution and mobile phase B: 0.1 % formic acid in acetonitrile. Primary and secondary mass spectrometric data were captured on the Waters Xevo G2-XS QTOF high-resolution mass spectrometer (Waters, USA) in MSe mode, managed by acquisition software (MassLynx V4.2, Waters). This data acquisition cycle enabled simultaneous dual-channel data collection for both low and high collision energies. The low collision energy was set at 2 V, while the high collision energy spanned from 10 to 40 V, with a scanning frequency of 0.2 s per mass spectrometer. Original data gathered via MassLynx V4.2 underwent processing using Progenesis QI software for peak extraction, alignment, and additional data operations. Identification relied on the online METLIN database incorporated into Progenesis QI software, along with public and self-built Baimaike databases. Simultaneous theoretical fragment identification was performed, the mass deviation of the parent ion was maintained within 100 ppm, while the fragment ion's mass deviation remained within 50 ppm (Wang et al., 2016).

### Screening of the differentially expressed genes and validation by real-time fluorescent quantitative PCR (RT-qPCR)

Total RNA extraction from testicular tissue utilized the Trizol method. The extracted RNA was then reverse transcribed to obtain cDNA according to the instructions of PrimeScript^TM^ RT reagent Kit (Takara Bio, Jiangsu, China)with cDNA Eraser. Sequencing analysis identified 6 differentially expressed genes: *ADH6, CYP11A1, CYP17A1, HSD3B1, FMO3*and *DHCR24*. Gene sequences were sourced from the jungle fowl in GenBank, with primers crafted using Prime 5.0 software (Table 2), and synthesized by Kunming Qingke Biotechnology Co, LTD. The quantitative cDNA, produced via reverse transcription, served as the template, and *GADPH* acted as the internal control. The reaction mixture was prepared per TB Green premix Ex Taq^TM^ Ⅱ quantitative kit(Takara Bio, Jiangsu, China) instructions. The reaction system comprised 20μL: 12.5μL (2 ×) TB Green premix Ex Taq Ⅱ (Tli RNase H Plus), 1μL each of upstream and downstream primers, 2μL of cDNA, and 8.5 μL of ddH_2_O. The PCR amplification protocol included a pre-denaturation phase at 95 °C for 30 s; the denaturation phase at 95°C for 5 s, followed by annealing at 60°C for 30 s (40 cycles). The melting curve process entailed 95 °C for 15 s, 60 °C for 1 min, and 95 °C for 15 s. Each sample underwent three biological replicates. Three biological replicates were performed for each sample.

**Table 2:** Primer information

gene name	primer sequence (5’-3’)	annealing temperature
*GAPDH*	F:GACAGCCATTCCTCCACCTT	59.0
	R:AACTGAGCGGTGGTGAAGAG	
*DHCR24*	F:AGACAGCGAGAGACAGGTTG	59.0
	R:CAGGTGTGTTGGAAAAGCCC	
*ADH6*	F:CACCTCACTGAACCACAAAACC	57.5
	R:GAGAAACCACAGGCAAACACAC	
*CYP11A1*	F: TCCGCCACCTCAACACCAAGA	59.0
	R:CACAAGGAGGCTGAAGAGGATGC	
*CYP17A1*	F: CGCATCCCTGTGTGAGA	60.0
	R: CAGCAGAGCCAAGTCCC	
*HSD3B1*	F:GCCAAAGAGGAGCAAACCAGAG	57.0
	R:TCCAGCAGTAAGCGAACGATCC	
*FMO3*	F: ATGAGGCTATCTGTTCCCAAAG	58.5
	R:GACCAATCCAATGACTGCCA	

### Data analysis

The hormone data underwent organization via Excel, with independent t-tests and significance analyses executed using SPSS 26.0. Statistical outcomes were presented as (mean ± standard deviation).

Transcriptomic data analysis employed the DESeq method using R software, applying | log2FC | > 1 and *P* ≤ 0.05 as screening criteria to identify differentially expressed genes (DEGs). Heat maps and volcanic plots illustrated the variations between the two groups, Subsequently, GO and KEGG analyses of DEGs were performed using the hypergeometric distribution in clusterProfiler. Gene expression calculations utilized the 2^−△△CT^ method, with GADPH serving as the internal reference.

For metabolomic analysis, R programming supported principal component analysis (PCA) and orthogonal partial least squares discriminant analysis (OPLS-DA) (Thévenot et al., 2015) modeling, implementing multiple cross-validation to calculate Variable Importance Projection (VIP) values. Differential Metabolites were selected based on fold change, P values, and VIP values from the OPLS-DA model. The criteria included FC > 1, *P* < 0.05, and VIP > 1. A hypergeometric distribution test was used to calculate the enrichment significance of differential Metabolites in the KEGG pathway.

Finally, a joint analysis of differentially expressed genes and Metabolites applied | CC | > 0.80 and CCP < 0.05 to define thresholds, followed by KEGG pathway enrichment analysis, with *P* < 0.05 indicating significant pathway enrichment.

## Results and discussion

### Effect of different light times on blood testosterone content

**Fig. 1:**
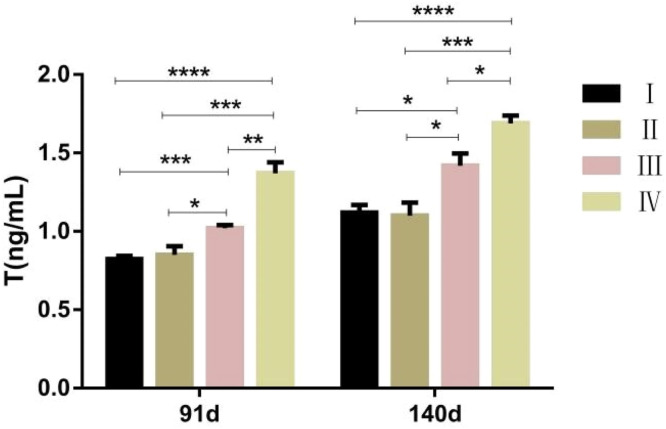
Effect of different light times on blood testosterone content.

Note: Ⅰ is the simulated natural light group, Ⅱ is the 12L:12D group, Ⅲ is the 16L:8D group, and Ⅳ is the 20L:4D group. * means *P* < 0.05, ** means *P* < 0.01, *** means *P* < 0.001, **** means *P* < 0.0001.

Fig. 1 illustrates that at 91 days of age, the Testosterone (T) levels in the blood of roosters in group IV were significantly elevated compared to those in groups I, II, and III (*P* < 0.01). Furthermore, group III exhibited significantly higher T levels than group I (*P* < 0.01) and group II (*P* < 0.05). At 140 days of age, group IV's blood T levels remained significantly higher than those in groups I and II (*P* < 0.01), as well as significantly surpassing group III (*P* < 0.05). Additionally, group III's blood T content was significantly elevated compared to groups I and II (*P* < 0.05). The findings indicate that Testosterone levels in the blood increase with extended exposure to light.

### Transcriptome sequencing results

#### Quality control of sequencing data

Only Raw Data was obtained after the data was offline, and the number of clean reads of each sample was more than 5.74 Gb by filtering the raw data, indicating that the amount of data was rich. Additionally, the percentage of Q20 bases in every sample remained above 97.86 %, while the percentage of Q30 bases exceeded 94.05 %, indicating high sequencing quality. The GC content and base quality exhibited a favorable distribution. Detailed data are presented in Table 3. These findings confirm that the Transcriptome sequencing data obtained in this study is of high quality and suitable for subsequent experiments and analyses.

**Table 3:** Quality control of mRNA sequencing data

Sample	Clean Reads	GC(%)	Q20(%)	Q30(%)
C-Tes1	41,451,518	48.76	98.14	94.75
C-Tes2	43,107,616	48.21	98.11	94.64
C-Tes3	38,794,800	48.17	98.07	94.52
C-Tes4	41,462,176	47.82	97.98	94.33
20-Tes1	39,948,590	47.90	97.91	94.16
20-Tes2	42,941,756	4 8.23	98.01	94.45
20-Tes3	42,917,490	47.97	97.86	94.05
20-Tes4	41,887,916	47.62	97.96	94.27

Note: C stands for simulated natural light group; 20 represents a 20-hour light group

#### Alignment analysis with the reference genome

By analyzing the reference genome, we observed that the alignment efficiency of Clean Reads across samples ranged from 94.22 % to 94.83 %. This suggests a high utilization of Transcriptome data. Furthermore, the proportion of Clean Reads aligned to unique positions in the reference genome for each sample ranged from 91.80 % to 92.52 %, indicating that the sequencing quality satisfied subsequent analytical requirements. Detailed alignment data appear in Table 4.

**Table 4:** Sequence alignment result

Sample	Total Reads	Mapped Reads	Uniq Mapped Reads	Multiple Map
C-Tes1	41,451,518	39,195,483 (94.56%)	38,269,363 (92.32%)	926,120 (2.23%)
C-Tes2	43,107,616	40,769,294 (94.58%)	39,713,384 (92.13%)	1,055,910 (2.45%)
C-Tes3	38,794,800	36,788,833 (94.83%)	35,893,834 (92.52%)	894,999 (2.31%)
C-Tes4	41,462,176	39,192,240 (94.53%)	38,215,432 (92.17%)	976,808 (2.36%)
20-Tes1	39,948,590	37,755,847 (94.51%)	36,812,572 (92.15%)	943,275 (2.36%)
20-Tes2	42,941,756	40,458,298 (94.22%)	39,421,161 (91.80%)	1,037,137 (2.42%)
20-Tes3	42,917,490	40,598,494 (94.60%)	39,598,604 (92.27%)	999,890 (2.33%)
20-Tes4	41,887,916	39,562,348 (94.45%)	38,553,196 (92.04%)	1,009,152 (2.41%)

Note: C stands for simulated natural light group; 20 represents a 20-hour light group

#### Screening of differentially expressed genes

**Fig. 2:**
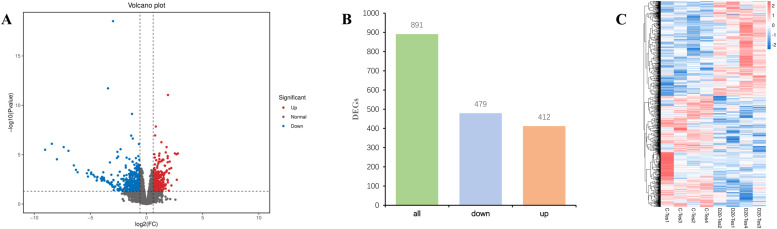
RNA-seq analysis (A) Volcano plot showed the DEGs; (B) DEGs number statistics; (C) Hierarchical cluster analysis of the DEGs.

As shown in Fig. 2, a total of 891 DEGs exhibited significant expression differences. Among them, 479 genes were significantly downregulated. These genes, which showed a marked reduction in expression under long photoperiods, are likely involved in biological processes that are no longer necessary or need to be suppressed under such light conditions. The extended photoperiod may have altered the organism's original physiological rhythms and metabolic demands, leading to the downregulation of certain genes to adapt to the new environmental conditions. Additionally, 412 genes exhibited a significant upregulation. These upregulated genes are biologically significant, as they likely encode key proteins in the light signal transduction pathway. Under long-day conditions, the light signal transduction pathway is activated, and to more effectively sense and respond to changes in light, the organism upregulates the expression of relevant genes to enhance the pathway's function, ensuring a timely and accurate response to light stimuli. The results from the clustering heatmap further revealed the gene expression differences between samples. The eight samples from the simulated natural light and 20 h light groups were clearly divided into two distinct branches. This marked clustering result suggests that genes within each cluster may share similar functional annotations or may be involved in the same metabolic pathways. This finding strongly indicates significant gene expression differences between the samples from different light treatment groups and reflects the profound impact of altered light conditions on gene expression regulation in the organism.

#### GO enrichment analysis of differentially expressed genes

**Fig. 3:**
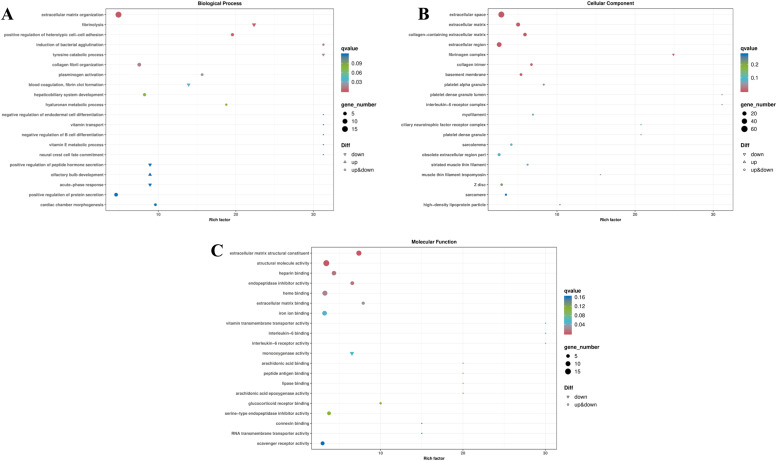
GO analysis of the DEGs; (A)DEGs enrichment to the top 20 items of biological processes; (B)DEGs enrichment to the top 20 items of cellular component; (C)DEGs enrichment to the top 20 items of molecular function.

Qvalue≤0.05 was used as the criterion for significant enrichment, and the most significantly enriched functional entries were selected from the results, which were plotted as GO enrichment bubbles as shown in Fig. 3, with a total of 24 significant trems enriched on biological processes, cellular components, and molecular functions. In biological processes, eight significant terms emerged, including extracellular matrix organization, fibrinolysis, positive regulation of heterotypic cell-cell adhesion, collagen fibril organization, tyrosine catabolic process, induction of bacterial agglutination, plasminogen activation, blood coagulation, and fibrin clot formation. In the cellular component category, ten significant terms were observed, consisting of extracellular space, extracellular matrix, collagen-containing extracellular matrix, extracellular region, fibrinogen complex, collagen trimer, basement membrane, interleukin-6 receptor complex, platelet dense granule lumen, and platelet alpha granule. Lastly, six significant terms were highlighted under molecular functions, namely extracellular matrix structural constituent, structural molecule activity, endopeptidase inhibitor activity, heparin-binding, heme binding, and extracellular matrix binding.

#### KEGG enrichment analysis of differentially expressed genes

**Fig. 4:**
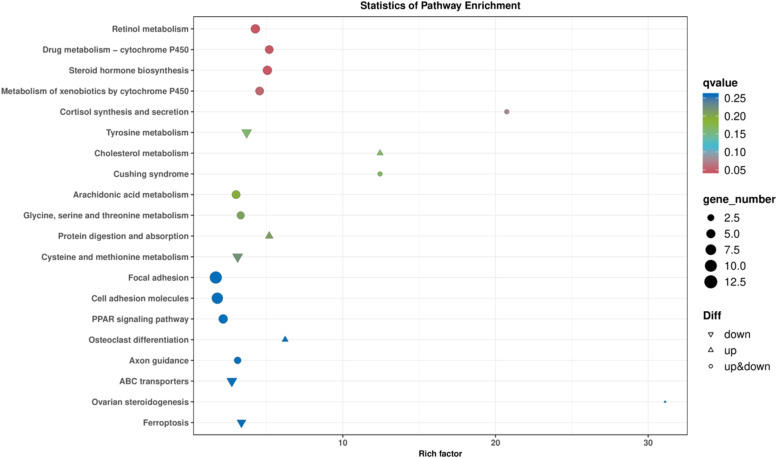
KEGG analysis of the DEGs.

The enrichment results were visualized using a bubble chart generated by ClusterProfiler, as illustrated in Fig. 4. A total of three significantly enriched signaling pathways were identified at the Qvalue ≤ 0.05 threshold: Steroid Hormone Biosynthesis, which enriched differentially expressed genes such as *CYP1A2, CYP11A1, CYP17A1*, and *HSD3B1*; drug metabolism via Cytochrome P450, which included differentially expressed genes like *HPGDS, ADH6*, and *FMO3*; and retinol metabolism, which enriched *CYP1A2, ALDH1A1*, and *ADH6*. Notably, both *CYP1A2* and *ADH6* were enriched in two signaling pathways.

### Untargeted metabolomics sequencing of testicular tissue

#### Quality control of sequencing data

**Fig. 5:**
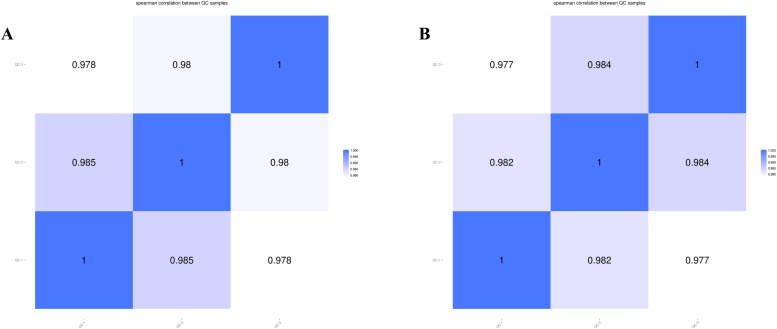
Correlation of metabolome QC samples; (A) Sample correlation in the negative ion mode; (B) Sample correlation in the positive ion mode.

If QC samples demonstrate high repeatability, then the instrument exhibits strong stability. A correlation coefficient exceeding 0.8 for QC samples confirms the instrument's reliability. Fig. 5 illustrates that the correlation coefficients for QC samples in both negative and Positive Ion Modes exceed 0.977. This finding signifies that the results from untargeted metabolomics sequencing in this study are dependable.

#### Annotation of metabolites

**Fig. 6:**
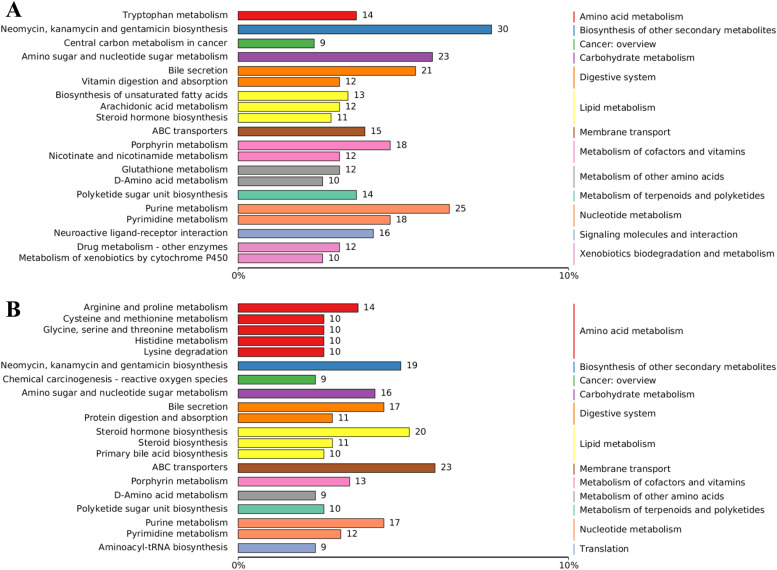
KEGG classification annotation of the metabolome; (A) Classification annotation of negative ion mode; (B) Classification annotation of positive ion mode.

We selected the top 20 annotations with the highest representation of KO pathway level 3 entries to create the bar chart shown in Fig. 6. For the Negative Ion Mode, we identified a total of 180 KO pathway level 3 entries, predominantly associated with metabolic pathways, followed closely by organic system pathways. The leading 10 KO pathway level 3 entries, annotated with the greatest number of Metabolites (Fig. 6A), included Neomycin, Kanamycin, Gentamicin biosynthesis, Purine metabolism, Amino sugar and nucleotide sugar metabolism, Bile secretion, Porphyrin metabolism, Pyrimidine metabolism, Neuroactive ligand-receptor interaction, ABC transporters, Tryptophan Metabolism, and Polyketide sugar unit biosynthesis.

In the Positive Ion Mode, we annotated 184 KO pathway level 3 entries, again mainly linked to metabolic pathways, followed by organic system pathways. The top 10 KO pathway level 3 entries with the highest number of Metabolites (Fig. 6B) featured ABC transporters, Steroid Hormone Biosynthesis, Neomycin, and the biosynthesis of Kanamycin and Gentamicin, along with Purine metabolism, Bile secretion, Amino sugar and nucleotide sugar metabolism, Arginine and Proline metabolism, and Steroid biosynthesis.

#### Orthogonal partial least squares discriminant analysis (OPLS-DA)

**Fig. 7:**
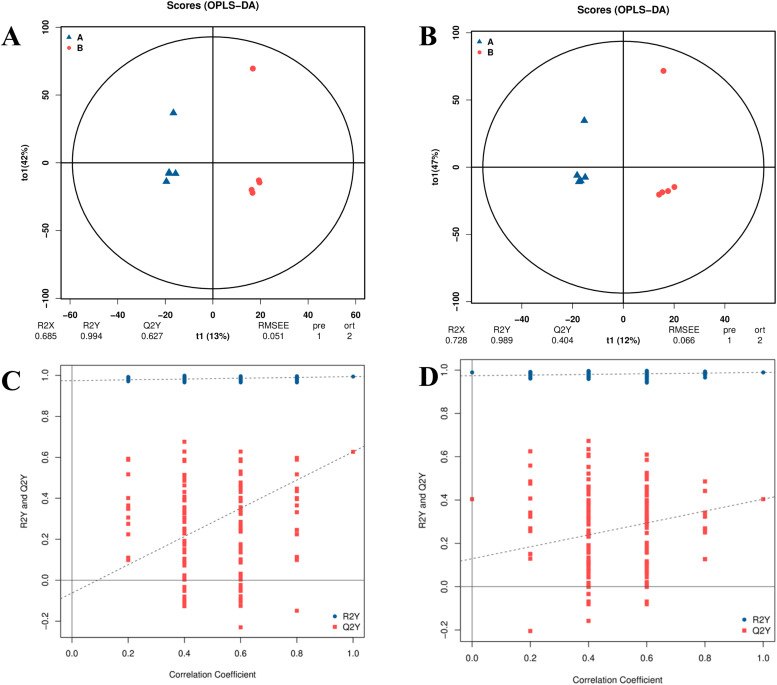
Scatter plot of OPLS-DA scores and replacement test of the metabolome; (A) Scatter plot of the score of negative ion mode; (B) Scatter plot of the score of positive ion mode; (C) Replacement test of negative ion mode; (D) Replacement test of positive ion mode.

If the slope of the regression line fitted by Q2Y is positive, it indicates that the model is meaningful, the blue dot is typically positioned above the red dot, suggesting a strong independence between the training set and the test set. According to the OPLS-DA score chart (Fig. 7A and B) for both negative and Positive Ion Modes, the samples from the two groups are positioned on the left and right sides of the circle, respectively. Sample points within each group are closely aggregated, while samples between the groups are more dispersed. The permutation test diagrams in both negative and Positive Ion Modes (Fig. 7C and D) further illustrate that the blue point is above the red point, confirming a positive slope for the Q2Y fitting regression line. This reinforces the conclusion that the model is meaningful and the data are reliable.

#### Differential metabolite analysis

Based on OPLS-DA results, we identified differential Metabolites by evaluating the Variable Importance Projection from the multivariate analysis model. We combined this with univariate analysis p-values or fold change values. The selection criteria included FC > 1, *P* < 0.05, and VIP > 1.

**Fig. 8:**
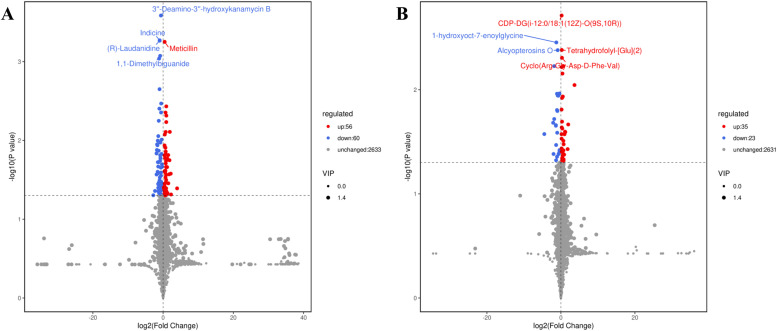
Volcanograms of differential metabolites; (A) Volcano map of negative ion mode; (B) Volcano map of positive ion mode.

**Fig. 9:**
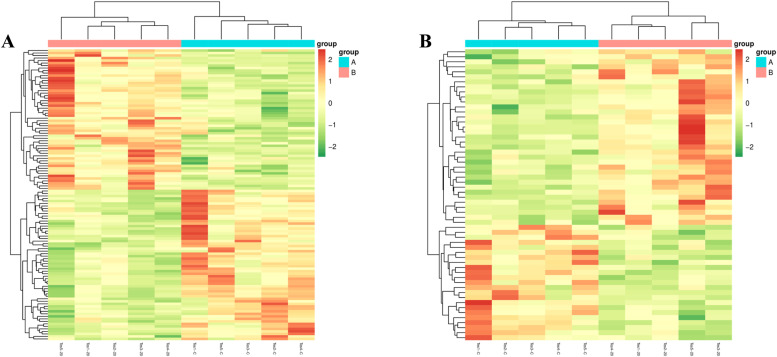
Clustering heat map of differential metabolites; (A) Clustering heat map ofnegative ion mode; (B) Clustering heat map of positive ion mode.

Fig. 8 demonstrates that in Negative Ion Mode, we identified 2,749 Metabolites in total. Among these, 116 Metabolites were distinguishable; notably, 56 exhibited upregulation, while 60 showed downregulation. The five most significant Metabolites included 3″-Deamino-3″-hydroxykanamycin B, Indicine, (R)-Laudanidine, Meticillin, and 1,1-dimethylbiguanide. In Positive Ion Mode, the analysis recognized 2,689 Metabolites, with 58 being distinguishable: 35 were upregulated, and 23 were downregulated. According to P-value rankings, the leading five Metabolites were cytidine diphosphate-diacylglycerol (i-12:0/18:1(12Z)-O(9S, *P* < 0.05)), 1-hydroxyoct-7-enoylglycine, Tetrahydrofolyl-[Glu](2), Alcyopterosins O, and Cyclo(Arg-Gly-Asp-d-Phe-Val);

Clustering heatmaps frequently illustrate clustering results. They convert a data matrix into various color gradients that represent numerical values. This representation corresponds to hierarchical clustering outcomes for both rows and columns. Fig. 9 displays unique clustering patterns for each differential metabolite in both negative and Positive Ion Modes, highlighting significant differences across the groups.

Metabolites may exhibit either synergistic or antagonistic interactions. For instance, similar trends in specific Metabolites suggest a positive correlation. In contrast, differing trends imply a negative correlation. We employed Pearson's correlation coefficient to measure these interactions, obtaining values near 1 for positive correlations and −1 for negative ones. We assessed the significance of these correlations using a p-value threshold of ≤ 0.05.

**Fig. 10:**
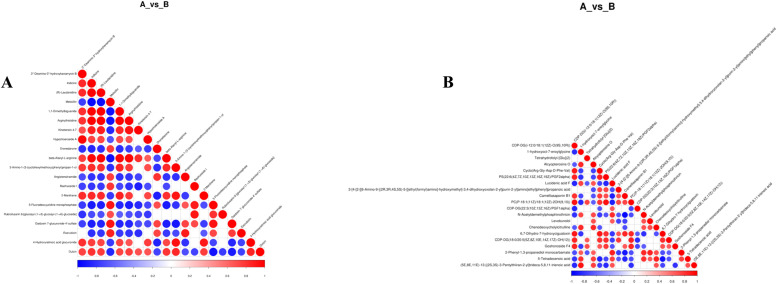
Correlation diagram of differential metabolites; (A) Correlation diagram of negative ion mode; (B) Correlation diagram of positive ion mode.

Fig. 10 demonstrates a strong positive correlation between (R)-laudanidine and indianine (*r* = 0.99) in Negative Ion Mode. It also reveals a significant negative correlation between methicillin and indianine (*r*=−0.95). Other notable correlations include 1,1-dimethylbiguanide with indiansine (*r* = 0.98) and (R)-laudanidine (*r* = 0.99). Furthermore, arginine-histidine displayed a positive correlation with Inosine (*r* = 0.98), (R)-laudanidine (*r* = 0.99), and 1,1-dimethyl metformin (*r* = 0.98). The analysis also uncovered significant positive correlations between Hypochondrioside A and 3″-deamine-3″‑hydroxy-kanamycin B (*r* = 0.97). β-alanyl-l-arginine positively correlated with indiansine (*r* = 0.96), (R)-laudanidine (*r* = 0.97), 1,1-dimethylbiguanide (*r* = 0.97), and arginine-histidine (*r* = 0.97). In the Positive Ion Mode, a notable positive correlation occurred between N-acetyl-Demethylphosphonate and Camelliaside B1 (*r* = 0.96).

#### KEGG enrichment analysis of differential metabolites

**Fig. 11:**
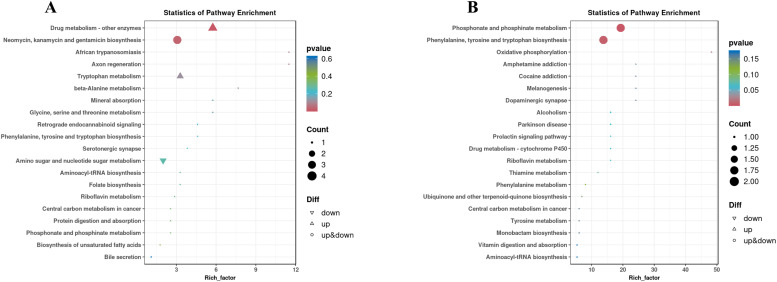
Differential metabolite KEGG enrichment bubble plots; (A) KEGG enrichment bubble plot in negative ion mode; (B) KEGG enrichment bubble plot in positive ion mode.

We conducted enrichment analysis on the KEGG annotation results of differential Metabolites and constructed a bubble map. Fig. 11 depicts the top 20 metabolic pathways enriched for differential Metabolites in both negative and Positive Ion Modes. In the Negative Ion Mode, the differential Metabolites primarily enrich metabolic pathways linked to amino sugar and nucleotide sugar metabolism, Tryptophan Metabolism, and various signaling pathways. In contrast, the Positive Ion Mode shows that differential Metabolites predominantly enrich in the biosynthesis of phenylalanine, tyrosine, and tryptophan. Additionally, they involve phosphonate and phosphinate metabolism, Cytochrome P450 (drug) metabolism, and steroid biosynthesis, among other signaling pathways.

### Combined analysis of transcriptome and metabolome of testicular tissue

#### Differential gene and differential metabolic analysis

**Fig. 12:**
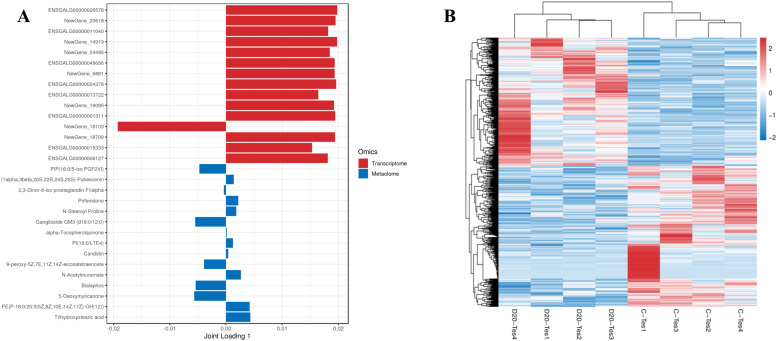
(A)Histogram of genes/metabolites with high O2PLS association and (B) Correlation analysis of differential genes and differential metabolites correlation coefficient matrix heat map and hierarchical clustering heat map.

**Fig. 13:**
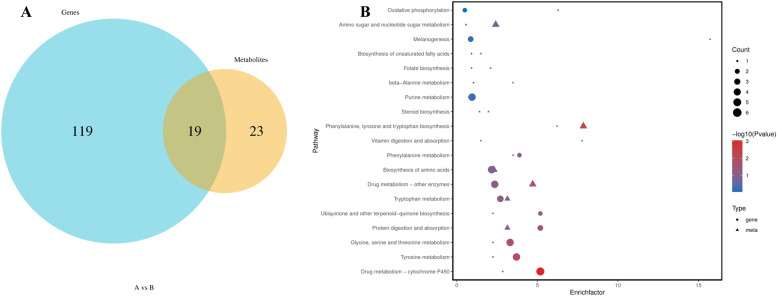
Venn diagram of differential genes and differential metabolite pathways (A) and KEGG enrichment bubble map of differential gene/metabolite significant enrichment pathway (B)

Fig. 12 illustrates the top 15 genes having the most significant effect on the Metabolome: ENSGALG00000026578, NewGene_20618, ENSGALG00000011040 (SCN3A), NewGene_14919, NewGene_24495, ENSGALG0000004968, NewGene_6881, ENSGALG00000024378 (ZNF414), ENSGALG00000013722, NewGene_19095, ENSGALG00000001311 (ARHGAP44), NewGene_18103, NewGene_18709, ENSGALG00000015333 (PCGF3), and ENSGALG00000006127 (FAM122A). Additionally, the top 15 Metabolites exhibiting the greatest impact on the Transcriptome include PIP (16:0/5 - iso PGF2VI), Pubescenin (1 alpha, beta 3, 20s, r, s, 24, 25s), 2, 3-Dinor-8-iso prostaglandin F1alpha, Pirfenidone, N-Stearoyl Proline, Ganglioside GM3 (d18:0/12:0), alpha-Tocopherolquinone, PI(18:0/LTE4), Candidin, 9-peroxy-5Z,7E,11Z,14Z-eicosatetraenoate, N-Acetylmuramate (n-acetofuryl ester), Bialaphos, 5-Deoxymyricanone, and PE(P-18:0/20:5(5Z,8Z,10E,14Z,17Z)-OH(12)). To effectively illustrate the expression pattern differences of significantly distinct genes and Metabolites, we conducted a hierarchical clustering analysis based on their expression levels. The results appear in the hierarchical clustering heatmap presented in Fig. 13B. When combined with the correlation coefficient matrix heat map and correlation analysis hierarchical clustering heat map, the analysis reveals a significant correlation between differential genes and Metabolites.

#### KEGG pathway enrichment analysis of differential genes and differential metabolites

Fig. 13A illustrates the identification of 19 signaling pathways enriched with differential genes and Metabolites. Fig. 13B lists these co-enriched pathways, which include: Drug Metabolism-Cytochrome P450, Tyrosine Metabolism, Glycine, Serine, and Threonine Metabolism, Protein Digestion and Absorption, Ubiquinone and Other Terpenoid Quinones Biosynthesis, Tryptophan Metabolism, and Drug Metabolism-Other Enzymes, Biosynthesis of Amino Acids, Phenylalanine Metabolism, Vitamin Digestion and Absorption, Biosynthesis of Phenylalanine, Tyrosine, and Tryptophan, Steroid Biosynthesis, Purine Metabolism, Beta-Alanine Metabolism, Folate Biosynthesis, and Biosynthesis of Unsaturated Fatty Acids, Melanogenesis, Amino Sugar and Nucleotide Sugar Metabolism, and Oxidative Phosphorylation. Notably, Drug Metabolism-Cytochrome P450 and Steroid Biosynthesis closely relate to animal sexual maturation.

**Fig. 14:**
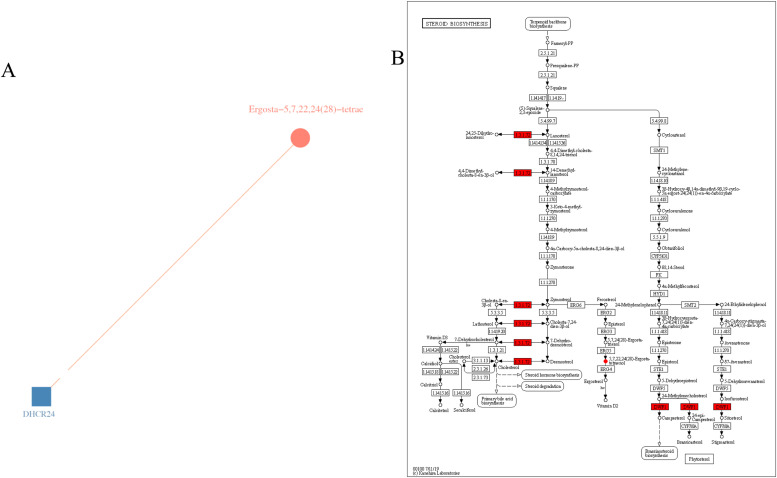
Correlation network of differential genes and metabolites with KEGG pathway analysis.

In the steroid biosynthesis signaling pathway, the differentially expressed gene DHCR24 and the differentially abundant metabolite Ergosta-5,7,22,24(28)-tetraen-3β-ol (Ergosta) are jointly enriched. We constructed a network diagram by selecting differentially expressed genes and Metabolites from this pathway based on correlation screening, providing a visual representation of the relationships between Metabolites and genes. In Fig. 14A, Metabolites are labeled in red, and their correlations are indicated by lines. As shown in Fig. 14B and Table 5, the differential gene DHCR24 and the differential metabolite Ergosta-5,7,22,24(28)-tetraen-3β-ol exhibit a significant correlation, which may suggest a functional connection between the two and potential biological pathways or regulatory mechanisms.

**Table 5:** Correlation Table of Differential Genes and Differential Metabolites in the Steroid Biosynthesis Pathway

geneName	metaName	PCC
*DHCR24*	Ergosta-5,7,22,24(28)-tetraen-3beta-ol	0.82

### Candidate genes were verified by RT-qPCR

**Fig. 15:**
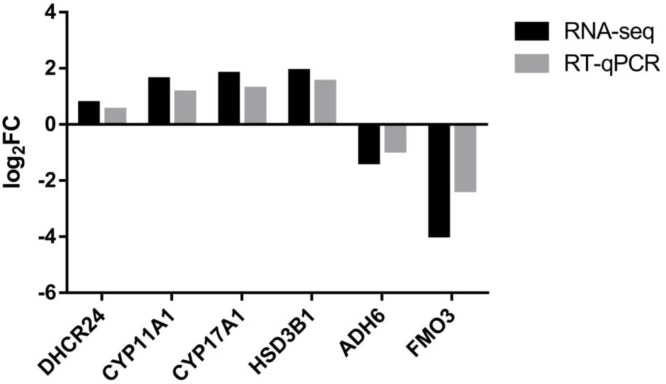
Validation results of transcriptome differential genes.

Through Transcriptome sequencing and integrated Transcriptome-Metabolome analysis, supplemented by GO enrichment and KEGG pathway analysis, we identified six candidate differentially expressed genes: C*YP11A1, CYP17A1, HSD3B1, ADH6, FMO3*, and *DHCR24*. Quantitative PCR experiments were conducted using RNA samples identical to those used in Transcriptome sequencing, with the results shown in Fig. 15. The gene expression levels obtained from quantitative PCR closely matched those from Transcriptome sequencing, providing strong evidence that the Transcriptome sequencing results are highly accurate, reliable, and reproducible.

## Discussion

Light significantly influences the reproductive performance of poultry by modulating the secretion of reproductive hormones via the hypothalamic-pituitary-gonadal axis. Tang (2013) demonstrated that in 90-day-old yellow-feathered broilers, Testosterone levels in roosters exposed to a 16 h light regimen exceeded those in the 23 h group. Yuan et al. (2019) explored the impact of various light durations (8L:16D, 12.5L:11.5D, 16L:8D) on the blood Testosterone levels of 20-week-old rabbits and found a marked increase in Testosterone correlating with extended light exposure. This study revealed that at 91 and 140 days, Testosterone levels in the 20 h light group were significantly higher than those in the simulated natural light, 12 h light, and 16 h light groups. Furthermore, the 16 h light group exhibited higher Testosterone levels compared to the simulated natural light and 12 h light groups. These findings align with previous studies, suggesting that variations may stem from differences in the age at which light exposure began, the specific light rhythm employed, and the chicken breeds involved.

Steroid hormone synthesis initiates with cholesterol, the sole precursor, which converts to pregnenolone. Subsequently, pregnenolone undergoes several enzymatic transformations, including hydroxylation, dehydrogenation, and cleavage, leading to the formation of various steroid hormones such as Testosterone, estradiol, and prolactin. The Steroid Hormone Biosynthesis pathway governs the production of FSH, LH, estrogen, Testosterone, and additional steroid hormones. Genes like *CYP11A1, CYP17A1*, and *HSD3B1*, which exhibit differential expression, enrich the Steroid Hormone Biosynthesis signaling pathway and may play a role in Testosterone secretion by Chahua No.2 roosters under light exposure. *CYP11A1* shows high expression in steroid-responsive tissues, including the testis, ovary, adrenal gland, and uterus, promoting Testosterone synthesis in male mouse testis (Ji et al., 2004). Yuan. (2022) experiments demonstrated that *CYP17A1* levels in follicular fluid positively correlate with TEST, DHEAS, and FTI, indicating the significant influence of the *CYP17A1* gene on androgen synthesis. *CYP11A1* impacts the conversion of sex hormones (estrogen and androgen) and is crucial in sex hormone regulation (Kaur et al., 2021). Emam et al. (2021). reported that flaxseed oil inhibited *CYP11A1* expression, leading to a marked decrease in Testosterone levels in rats. Hirsch et al. (2012) revealed that metformin could suppress androgen synthesis by affecting *CYP17A1* activity. *HSD3B1* plays a vital catalytic role in steroid hormone synthesis, converting low-activity steroids into high-activity hormones like Testosterone and progesterone through dehydrogenation (Gao et al. ,2019). Experimental studies by Dziendzikowska et al. (2016), Liu et al. (2016), Li et al. (2016) found that inhibition of *HSD3B1* significantly inhibited androgen synthesis, resulting in delayed gonadal development and reproductive issues, including reduced gonadal capacity in testes and ovaries, diminished reproductive ability, and abnormal reproductive organs. Lavoie et al. (2009) study confirmed that a lack of *HSD3B1* leads to decreased aldosterone and cortisol synthesis along with reduced Testosterone synthesis. In this study, the expression of *CYP11A1*, CYP17A1, *HSD3B1*, and other genes significantly increased in the 20 h light exposure group. Combined with the above literature, the content of Testosterone should be significantly increased in the 20 h light exposure group, which is consistent with the results of this study. These results imply that light may regulate Testosterone synthesis and secretion by modulating the expression of *CYP11A1, CYP17A1, HSD3B1*, and other related genes.

The Cytochrome P450 pathway plays a crucial role in the metabolism of steroid sex hormones like estrogen, Testosterone, FSH, and LH, as highlighted in this study. Cytochrome P450 (CYP or CYP450) refers to a group of enzymes that are critical for the synthesis and metabolism of both endogenous and exogenous substances, including steroid hormones and drugs. Research indicates that CYP-dependent oxygenation is a vital step in androgen synthesis signaling (Cederbaum et al., 2015). The CYP450 family comprises ten categories, classified based on their electron transfer and catalytic activities. The first category encompasses enzymes that facilitate steroid compound reactions (Hannemann et al., 2007). Noteworthy enzymes, such as *CYP11A1, CYP17A1, CYP3A4*, and *CYP3A5*, play significant roles in Testosterone synthesis. *CYP11A1* and *CYP17A1* exhibit high expression levels in the testes and other gonadal tissues. *CYP11A1* converts cholesterol into pregnenolone via a multi-step process, while *CYP17A1* catalyzes further transformations of pregnenolone (Miller and Auchus, 2011; Chien et al., 2017). Furthermore, *CYP3A4* oxidizes Testosterone into low-activity Metabolites. Notably, studies show that inhibiting CYP3A4 can markedly enhance Testosterone activity within cells (Nabid et al., 2018; Fujimura et al., 2009). Moilanen et al. (2007) demonstrated that compromise of *CYP3A5* activity can diminish androgen receptor (AR) levels in cell experiments. KEGG enrichment analysis of differentially expressed genes within the Cytochrome P450 signaling pathway identified genes like *HPGDS, ADH6*, and *FMO3*. The *ADH6* gene encodes alcohol dehydrogenase 6, an enzyme involved in ethanol metabolism, wherein ethanol oxidizes to acetaldehyde. It contains a 5′-noncoding region, which serves as a glucocorticoid response element overlapping the steroid hormone receptor binding site (Rodriguez et al., 2000). This suggests a potential influence of the *ADH6* gene on Testosterone secretion. Meanwhile, the *FMO3* gene encodes Flavin-containing Monooxygenase 3 (*FMO3*), and its activation leads to the production of Trimethylamine oxide (TMAO). TMAO is implicated in dysregulated cholesterol metabolism (Jia et al., 2021). Evidence from Zhang et al. (2022) suggests that TMAO may hinder cholesterol transport, resulting in increased cholesterol levels in the body. Notably, the expression of *FMO3* is significantly downregulated under 20 h light exposure, indicating that light exposure may dampen *FMO3* expression, thereby reducing TMAO production, enhancing cholesterol metabolism, and subsequently promoting Testosterone secretion.

The steroid biosynthesis signaling pathway exhibited enrichment through differential genes and Metabolites identified in the combined Transcriptome and Metabolome analysis. The *DHCR24* gene emerged as a significant differential gene, while Ergosta-5,7,22,24(28)-tetraene-3beta-ol surfaced as the notable differential metabolite. *DHCR24* functions as a 24-dehydrocholesterol reductase, facilitating the final step of cholesterol synthesis, which involves converting desosterols into cholesterol (Luu et al., 2013; 2015; Waterham et al., 2001). This gene is universally expressed in all cholesterol-synthesizing cells and tissues, with pronounced expression observed in certain cells or organs that produce steroid hormones, such as the testis. The differential metabolite, Ergosta-5,7,22,24(28)-tetraen-3beta-ol, is a derivative of ergosterol, a compound bearing structural similarities to cholesterol, that effectively mitigates cholesterol activity (Yang et al., 2023). In this investigation, expression levels of the *DHCR24* gene and Ergosterol-derived 5,7,22,24(28)-tetraen-3beta-alcohol experienced significant up-regulation in the 20 h light exposure group, which subsequently influenced cholesterol content within the testis as well as the synthesis and secretion of Testosterone in the subject animals.

## Conclusion

This study demonstrates that increasing light duration correlates with elevated Testosterone levels secreted by the testis. Through a comprehensive analysis of transcriptomic and metabolomic data, we examined how varying light durations influence Testosterone secretion in Chahua No.2 roosters. Our findings highlight the involvement of signaling pathways such as steroid hormone synthesis, steroid synthesis, and Cytochrome P450 in mediating the effects of light duration on Testosterone secretion. We elucidated transcriptional and metabolic patterns linked to this regulation, identifying key genes like C*YP11A1, CYP17A1, HSD3B1, ADH6, FMO3*, and *DHCR24*, alongside Metabolites such as Ergosterol-5,7,22,24 (28)-tetraene-3beta-alcohol. This research significantly enhances our understanding of the molecular mechanisms through which light duration impacts Testosterone secretion in Chahua No.2 roosters.

## Ethics approval

The experiments were performed following the Regulations for the Administration of Laboratory Animals and approved by the Life Science Ethics Committee of Yunnan Agricultural University (license number: APYNAU202412003).

## Declaration of competing interest

The authors declare that they have no known competing financial interests or personal relationships that could have appeared to influence the work reported in this paper.
